# Defects in Very Long Chain Fatty Acid Synthesis Enhance Alpha-Synuclein Toxicity in a Yeast Model of Parkinson's Disease

**DOI:** 10.1371/journal.pone.0015946

**Published:** 2011-01-11

**Authors:** Yong Joo Lee, Shaoxiao Wang, Sunny R. Slone, Talene A. Yacoubian, Stephan N. Witt

**Affiliations:** 1 Department of Biochemistry and Molecular Biology, Louisiana State University Health Sciences Center at Shreveport, Shreveport, Louisiana, United States of America; 2 Department of Neurology, Center for Neurodegeneration and Experimental Therapeutics, University of Alabama at Birmingham, Birmingham, Alabama, United States of America; National Institutes of Health, United States of America

## Abstract

We identified three *S. cerevisiae* lipid elongase null mutants (*elo1*Δ, *elo2*Δ, and *elo3*Δ) that enhance the toxicity of alpha-synuclein (α-syn). These elongases function in the endoplasmic reticulum (ER) to catalyze the elongation of medium chain fatty acids to very long chain fatty acids, which is a component of sphingolipids. Without α-syn expression, the various *elo* mutants showed no growth defects, no reactive oxygen species (ROS) accumulation, and a modest decrease in survival of aged cells compared to wild-type cells. With (WT, A53T or E46K) α-syn expression, the various *elo* mutants exhibited severe growth defects (although A30P had a negligible effect on growth), ROS accumulation, aberrant protein trafficking, and a dramatic decrease in survival of aged cells compared to wild-type cells. Inhibitors of ceramide synthesis, myriocin and FB1, were extremely toxic to wild-type yeast cells expressing (WT, A53T, or E46K) α-syn but much less toxic to cells expressing A30P. The elongase mutants and ceramide synthesis inhibitors enhance the toxicity of WT α-syn, A53T and E46K, which transit through the ER, but have a negligible effect on A30P, which does not transit through the ER. Disruption of ceramide-sphingolipid homeostasis in the ER dramatically enhances the toxicity of α-syn (WT, A53T, and E46K).

## Introduction

Alpha-synuclein (α-syn) is a neuronal protein that has been linked to Parkinson's disease (PD) by biochemical and genetic studies [1]. Alterations in α-syn cause the degeneration of dopaminergic neurons in an area of the mid-brain called the substantia nigra pars compacta [2], [3]. Loss of these neurons results in slowness of movement, resting tremor, rigidity, and disturbances of gait and posture [1], [4], [5], which are the clinical manifestations of PD. PD is but one of a group of alpha-synucleinopathies that also consists of dementia with Lewy bodies, multiple system atrophy and neurodegeneration with brain iron accumulation type 1.

Human molecular genetic studies have implicated many genes (*ATP13A2*, *DJ-1*, *HTRA2*, *LRRK2*, *MAPT*, *Parkin*, *PINK1*, and *UCH-L1*) [6], [7] other than α-syn [8], [9], [10], [11] that when mutated produce the constellation of symptoms known as PD. However, only 10% of all PD cases are due to specific mutations in these genes, whereas 90% of the cases are “sporadic”, meaning there is no known cause, although α-syn probably is connected to these cases. Environmental risk factors such as exposure to heavy metals or pesticides may make α-syn more toxic. This study has used yeast to identify other potential risk factors that can enhance the toxicity of α-syn. The unifying concept is that Parkinson's disease occurs in some individuals but not others because of an age-related decrement in the mechanisms that naturally protect cells from toxic alterations in α-syn.

α-Syn is a small (140 amino acids) protein that constitutes as much as 1% of the protein in human neurons. α-Syn has been proposed to regulate cell differentiation, synaptic plasticity and dopaminergic neurotransmission [12]. A defining feature of α-syn is its amazing ability to self-associate into an array of soluble and insoluble high molecular mass species [13], and various post translation modifications of α-syn can occur (dopamine adduct formation [14], phosphorylation [15], proteolysis [16], and nitration [17]) that can alter its aggregation behavior and thus toxicity. The insoluble β-sheet rich fibers of α-syn that accumulate in intracytoplasmic deposits, termed Lewy bodies, were once thought to be the toxic species, but this has fallen out of favor. Instead, evidence is mounting that the toxic α-syn species is a soluble high molecular mass protofibril that binds to and pokes holes in cellular membranes [18]. α-Syn can also cause proteasome dysfunction [19], [20], block endoplasmic reticulum (ER)-Golgi traffic [21], and inhibit histone acetylation [22], and α-syn mutants (A30P and A53T) block chaperone-mediated autophagy [23]. Given these many possibilities, teasing out the precise mechanism of α-syn toxicity has been challenging.

Genetic screens [21], [24], [25] have identified many genes that modify the toxicity of α-syn, but one of the most intriguing findings is that genes involved in lipid metabolism and vesicle trafficking preferentially modify the toxicity of α-syn [24]. In this study we discovered three lipid elongase genes (*ELO1*, *ELO2*, and *ELO3*) in *S. cerevisiae* that when deleted dramatically increase the toxicity of (WT, A53T, and E46K) α-syn but not of A30P, resulting in growth defects, aberrant trafficking, and ROS accumulation. Each of these elongases is a membrane bound protein that resides in the endoplasmic reticulum.

Elo1p elongates C14 fatty acids to C16 fatty acids [26]. Elo2p elongates C16/C18 fatty acids to C22/C24 [27], and Elo3p elongates C22/C24 fatty acids to C26 fatty acids [27], which are incorporated into C26-phytoceramide, a component of sphingolipids [28], [29]. Null mutations in *ELO2* or *ELO3* decrease sphingolipid levels and increase the level of the long chain base phytosphingosine [27]. Recent reports showed that *elo3*Δ cells cannot synthesize C26-fatty acids and C26-phytoceramide, and such cells by necessity produce ceramides and sphingolipids that contain C22 and C24 fatty acids (instead of C26-fatty acids) [30], [31]. The slightly altered ceramide/sphingolipid structure produces a severe trafficking defect at 37°C in which the essential plasma membrane H+-ATPase proton pump Pma1p fails to oligomerize in the ER [32] and fails to traffic to the plasma membrane [31]; instead, it mistargets to the vacuole. The results in this report reveal that disruption of the lipid-sphingolipid homeostasis in the ER dramatically increases the toxicity of α-syn.

## Results

The strains and plasmids used in this study are given in Table 1. A targeted screen of nineteen strains from the yeast deletion collection in which the genes deleted are involved in lipid metabolism was conducted (Table 2). Strains were transformed with a high copy plasmid containing α-syn under control of the gal promoter, serially diluted on plates with inducing media (+gal), and scored for growth inhibition compared to the uninduced condition (-gal). This approach led to the identification of six non-essential genes that when deleted enhance the toxicity of α-syn toxicity: *ISC1*, *ELO1*, *ELO2*, *ELO3*, *OSH1*, and *OSH2*. The latter two genes were recently reported to modify the toxicity of α-syn [33]. Here, we focused on characterizing the *ELO* genes that code for membrane-bound enzymes of the endoplasmic reticulum (ER) that elongate medium chain fatty acids to C26-VLCFA. Given the importance of lipid metabolism genes and vesicle trafficking genes in regulating α-syn toxicity [24], we analyzed the effects of *elo* deletions on yeast expressing the various α-syns (WT, A30P, A53T, and E46K) using a variety of assays.

**Table 1:** *S. cerevisiae* strains and plasmids.

Strain and plasmid	Description	Source
BY4741	*MAT*a *his3Δ1 leu2Δ0 met15Δ0 ura3Δ0*	ATCC
BY4741-ELO1	*elo1Δ::KanMX MAT*a *his3Δ1 leu2Δ0 met15Δ0 ura3Δ0*	ATCC
BY4741-ELO2	*elo2Δ::KanMX MAT*a *his3Δ1 leu2Δ0 met15Δ0 ura3Δ0*	ATCC
BY4741-ELO3	*elo3Δ::KanMX MAT*a *his3Δ1 leu2Δ0 met15Δ0 ura3Δ0*	ATCC
BY4741-LAC1	*lac1Δ::KanMX MAT*a *his3Δ1 leu2Δ0 met15Δ0 ura3Δ0*	ATCC
BY4741-LAG1	*lag1Δ::KanMX MAT*a *his3Δ1 leu2Δ0 met15Δ0 ura3Δ0*	ATCC
SC288C-ISC1	*isc1Δ::KanMX MAT*α *his3Δ1 leu2Δ0 met15Δ0 ura3Δ0*	Kelly Tatchell
BY4741-IFA38	*ifa38Δ::KanMX MAT*a *his3Δ1 leu2Δ0 met15Δ0 ura3Δ0*	ATCC
BY4741-PDR1	*pdr1Δ::KanMX MAT*a *his3Δ1 leu2Δ0 met15Δ0 ura3Δ0*	ATCC
SC288C-PDR3	*pdr3Δ::KanMX MAT*α *his3Δ1 leu2Δ0 met15Δ0 ura3Δ0*	Kelly Tatchell
SC288C-YDC1	*ydc1Δ::KanMX MAT*α *his3Δ1 leu2Δ0 met15Δ0 ura3Δ0*	Kelly Tatchell
SC288C-LCB3	*lcb3Δ::KanMX MAT*α *his3Δ1 leu2Δ0 met15Δ0 ura3Δ0*	Kelly Tatchell
SC288C-LCB4	*lcb4Δ::KanMX MAT*α *his3Δ1 leu2Δ0 met15Δ0 ura3Δ0*	Kelly Tatchell
SC288C-PCT1	*pct1Δ::KanMX MAT*α *his3Δ1 leu2Δ0 met15Δ0 ura3Δ0*	Kelly Tatchell
SC288C-CWH43	*cwh43Δ::KanMX MAT*α *his3Δ1 leu2Δ0 met15Δ0 ura3Δ0*	Kelly Tatchell
SC288C-EEB1	*eeb1Δ::KanMX MAT*α *his3Δ1 leu2Δ0 met15Δ0 ura3Δ0*	Kelly Tatchell
SC288C-YSR3	*ysr3Δ::KanMX MAT*α *his3Δ1 leu2Δ0 met15Δ0 ura3Δ0*	Kelly Tatchell
SC288C-FOX1	*fox1Δ::KanMX MAT*α *his3Δ1 leu2Δ0 met15Δ0 ura3Δ0*	Kelly Tatchell
SC288C-AYR1	*ayr1Δ::KanMX MAT*α *his3Δ1 leu2Δ0 met15Δ0 ura3Δ0*	Kelly Tatchell
BY4741-SUR2	*sur2Δ::KanMX MAT*a *his3Δ1 leu2Δ0 met15Δ0 ura3Δ0*	ATCC
SC288C-OSH1	*osh1Δ::KanMX MAT*α *his3Δ1 leu2Δ0 met15Δ0 ura3Δ0*	Kelly Tatchell
SC288C-OSH2	*osh2Δ::KanMX MAT*α *his3Δ1 leu2Δ0 met15Δ0 ura3Δ0*	Kelly Tatchell
BY4741-ELO1-TAP	TAP-tagged BY4741 at *ELO1* C-terminal	Open Biosystems
pAG426GAL	2* µ URA3 Amp* ^r^ *GAL1* promoter	Addgene
pAG426GAL-EGFP	EGFP in pAG426GAL	Addgene
pAG415GPD	Low copy *CEN LEU2 ARS Amp* ^r^ *GPD1* promoter	Addgene
pAG425GAL	2* µ LEU2 Amp* ^r^ *GAL1* promoter	Addgene
pAG415GPD-TAP	TAP in pAG415GPD	Addgene
pAG425GAL-TAP	TAP in pAG425GAL	Addgene
pAG426GAL-WT	WT α-syn in pAG426GAL	This study
pAG426GAL-A30P	A30P α-syn in pAG426GAL	This study
pAG426GAL-A53T	A53T α-syn in pAG426GAL	This study
pAG426GAL-E46K	E46K α-syn in pAG426GAL	This study
pAG426GAL-EGFP-WT	WT α-syn in pAG426GAL-EGFP	This study
pAG415GPD-ELO1	*ELO1* in pAG415GPD	This study
pAG415GPD-ELO1-TAP	*ELO1* in pAG415GPD-TAP	This study
pAG425GAL-ELO1	*ELO1* in pAG425GAL	This study
pAG425GAL-ELO1-TAP	*ELO1* in pAG425GAL-TAP	This study
pAG426GAL-EGFP-mts1	EGFP-mts1 in pAG426GAL	This study
pAG426GAL-EGFP-mts2	EGFP-mts2 in pAG426GAL	This study

**Table 2:** Analysis of the growth of wild-type (BY4741) and deletion mutants with or without WT α-syn.

	Growth^b^	
Strain^a^	− α-syn	+ α-syn
WT	+++	++
*lac1*Δ	+++	++
*lag1*Δ	+++	++
*isc1*Δ	+++	+
*ifa38*Δ	+++	++
*ydc1*Δ	+++	++
*lcb3*Δ	+++	++
*lcb4*Δ	+++	++
*elo1*Δ	+++	+
*elo2*Δ	+++	+
*elo3*Δ	+++	+
*pct1*Δ	+++	++
*cwh43*Δ	+++	++
*eeb1*Δ	+++	++
*ysr3*Δ	+++	++
*fox1*Δ	+++	++
*ayr1*Δ	+++	++
*sur2*Δ	+++	++
*osh1*Δ	+++	+
*osh2*Δ	+++	+

a Strains deleted for these genes are listed in Table 1.

b Strains harboring empty vector or WT α-syn were spotted onto SC-galactose medium. Growth was scored on a scale from +++ > ++ > + after 3 days at 30°C.

### Null mutations of fatty acid elongase genes enhances the toxicity of α-syn

**Figure 1:**
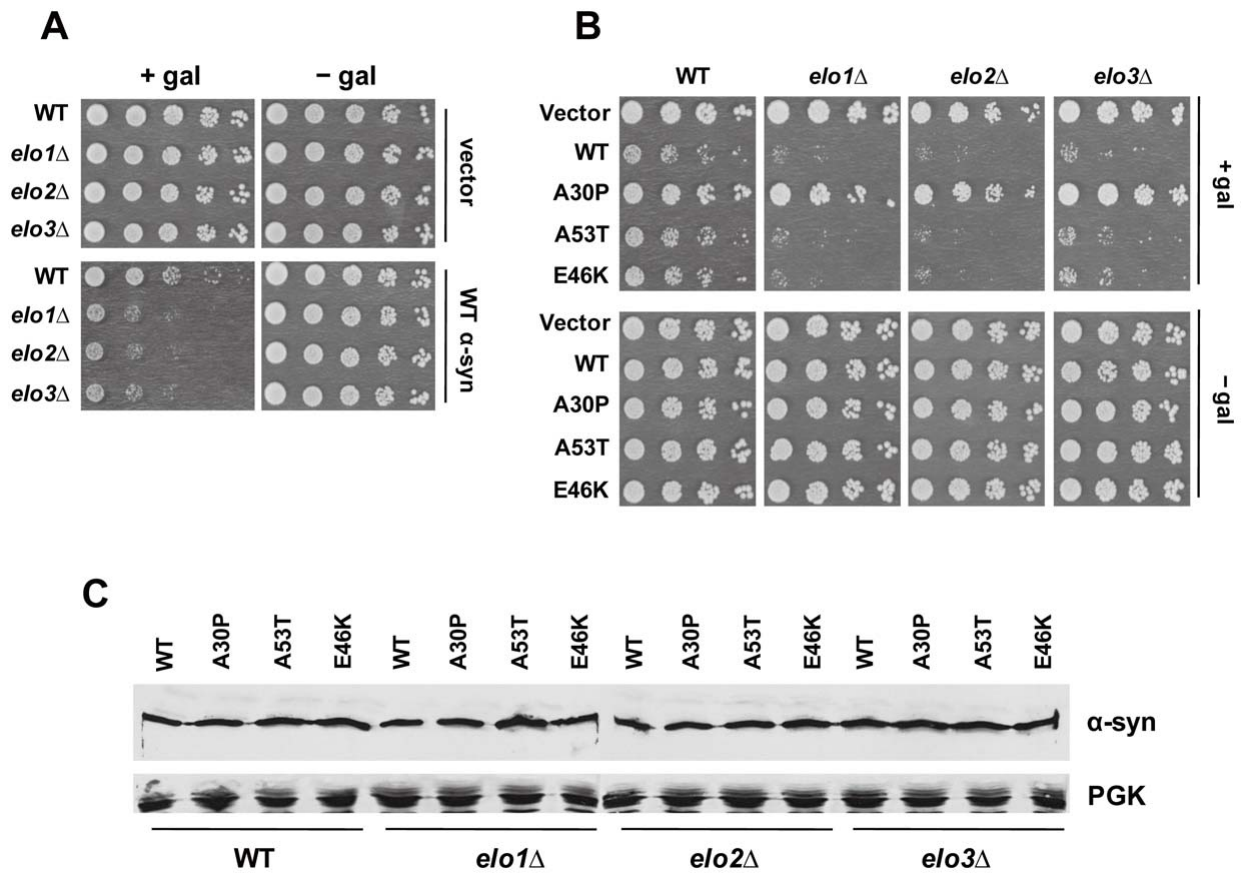
Enhanced α-syn toxicity in elongase null mutants. (A) The effect of WT α-syn expression on the growth of three deletion strains (*elo1*Δ, *elo2*Δ, and *elo3*Δ) and the wild-type BY4741 strain was evaluated. In panels (A, B), strains were transformed with pAG426GAL-WT (WT α-syn), pAG426GAL-A30P (A30P), pAG426GAL-A53T (A53T), pAG426GAL-E46K (E46K) or pAG426GAL (empty vector) and spotted in successive 10-fold serial dilutions on solid sucrose and solid galactose plates and incubated for 3 days at 30°C. Experiments were repeated three times with similar results. (B) The effect of the various α-syns (WT, A30P, A53T, or E46K) on the growth of the three *elo* deletion strains was also evaluated. (C) Western blot analysis of yeast cells expressing α-syn. Lysates were prepared from cultures grown for 6 h in inducing medium and then subjected to SDS–PAGE followed by western blot analysis. The cell-signaling polyclonal antibody against α-syn was used to visualize the three α-syns. The loading control was the yeast protein Pgk1p. Identical amounts of protein were loaded per well. Plasmids: see panel (A).

The growth of the *elo* null mutants, with or without WT α-syn, compared to growth of the wild-type strain (BY4741) is shown Fig. 1A. Without α-syn expression, the three *elo* mutants showed the same growth as the wild-type strain. In contrast, with WT α-syn expression, each *elo* mutant grew much slower than the wild-type strain. The results show that loss of any one of the three elongase enzymes increases the toxicity of WT α-syn.

The α-syn variants associated with early-onset PD, *i.e.*, A30P, A53T, and E46K were also tested in the growth assay. The *elo1*Δ strain expressing A53T, E46K, or WT α-syn showed very slow growth compared to the same cells with empty vector (Fig. 1B); whereas, *elo1*Δ cells expressing A30P grew similar to the same cells with empty vector. The same pattern was observed for the other two deletion strains, *elo2*Δ and *elo3*Δ, in that these two strains showed a synthetic sick phenotype with A53T, E46K, and WT α-syn but not with A30P. Western blot analysis demonstrated similar expression levels for the various α-syns in the various strains (Fig. 1C); thus, the lack of an effect of A30P is not due to low expression level. Because the three Elo enzymes are integral membrane proteins that reside in endoplasmic reticulum, deleting any one of the three elongase enzymes might preferentially affect the classical secretory pathway. WT α-syn and A53T [34], and probably E46K because it associates with the plasma membrane, transit through the secretory pathway in yeast and mammalian cells [34], [35], whereas, A30P does not. The results show that the three elongase null mutants exhibit synthetic sick phenotypes with only those α-syns (WT, A53T, and E46K) that transit through the secretory pathway.

**Figure 2:**
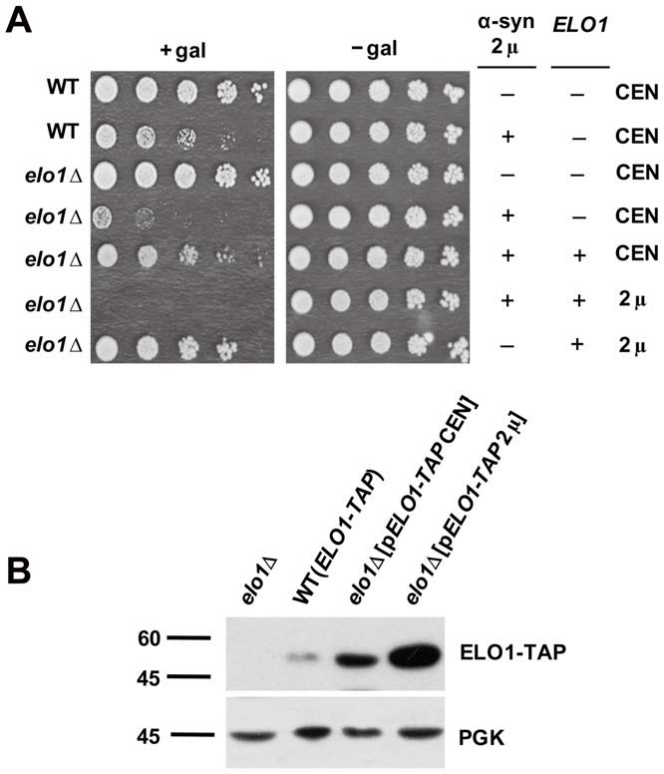
*elo1*Δ/+α-syn slow growth phenotype is rescued by *ELO1*. (A) Growth properties of wild-type cells and *elo1*Δ cells expressing WT α-syn (pAG426GAL-WT) and expressing Elo1p from a CEN plasmid (pAG415GPD-ELO1) or over expressing from a 2 µ plasmid (pAG425GAL-ELO1 are shown. The spot assay was conducted as described in Fig. 1. (B) The western blot analysis shows the level of Elo1p-Tap protein in ELO1-TAP cells, in *elo1*Δ cells transformed with pAG415GPD-ELO1 (CEN, *GPD1* promoter), and in *elo1*Δ cells transformed with pAG425GAL-ELO1 (2 µ, *GAL1* promoter). A monoclonal antibody against the tap tag was used, and Pgk1 was used as the loading control.

We also sought to determine whether the *elo1*Δ/+α-syn slow growth phenotype could be rescued by a plasmid carrying wild-type *ELO1* (Fig. 2A). A low-copy plasmid (CEN) carrying wild-type *ELO1* rescued the slow growth phenotype (compare row 5 to rows 2 and 4), whereas a high copy plasmid (2 µ) carrying wild-type *ELO1* was lethal (row 6). Over- expression of Elo1p from the high copy plasmid in *elo1*Δ/-α-syn cells was also slightly toxic compared to wild-type cells (compare row 7 to row 1). To verify that Elo1p was over- expressed, western blot analysis showed the highest level of the Elo1p-Tap enzyme in the *elo1*Δ strain with the high copy plasmid, then the *elo1*Δ strain with the low copy *ELO1* plasmid, and then the ELO1-TAP integrated strain (with its natural promoter) (Fig. 2B). Many genes when over-expressed cause toxicity, and this has been reported for *ELO1* [36]. The data suggests that the toxicity of α-syn exhibits a U-shaped toxicity curve with respect to Elo1p/palmitate.

We asked whether exogenous medium chain fatty acids could protect *elo1*Δ cells from α-syn. Different length medium chain fatty acids (from C12 to C18) and phytosphingosine (PHS) were tested in *elo1*Δ cells. The addition of these fatty acids into the medium caused turbidity; thus, instead of monitoring optical density, which detects live and dead cells and is affected by turbidity, aliquots of the culture were removed at the indicated time, diluted, and spread over plates containing rich media. The number of colony forming units (CFUs) was obtained in this way.

Yeast media supplemented with medium chain fatty acids at a low concentration (1 mM) failed to protect the BY4741 wild-type cells from WT α-syn, whereas at a ten-fold higher concentration (10 mM) certain fatty acids (C16, C18, and PHS) even increased WT α-syn toxicity (Table 3). Similar experiments were conducted on *elo1*Δ cells, and for these cells the toxicity of α-syn is readily seen by the precipitous 90% drop in CFUs upon expression of α-syn: 110 ±6×10^6^ cfu/ml (-αS) → 12 ±8×10^6^ cfu/ml (+αS) (top row, Table 3). C16 palmitic acid, C18 stearic acid and PHS at low concentration (1 mM) partially rescued the toxicity of WT α-syn in *elo1*Δ cells, as judged by the 4-5-fold increase in CFUs, and increasing the fatty acid concentration failed to give additional protection. Because Elo1p catalyzes the formation of C16 palmitic acid, it was expected that palmitic would be beneficial to *elo1*Δ cells, which is what was found.

**Table 3:** Analysis of exogenous fatty acids on the growth of wild-type cells and *elo1*Δ cells with or without WT α-syn.

	WT (cfu × 10^6^/ml)		*elo1*Δ (cfu × 10^6^/ml)	
compound	vector	WT α-syn	vector	WT α-syn
(−)	126±14	74±13	110±16	12±8
12:0 (1 mM)	126±14	73±9	115±8	15±10
12:0 (10 mM)	112±10	58±8	105±9	9±3
16:0 (1 mM)	124±12	80±16	120±8	56±9
16:0 (10 mM)	104±11	21±11	119±10	20±5
18:0 (1 mM)	123±9	81±10	120±7	64±7
18:0 (10 mM)	102±7	28±6	107±12	17±6
PHS (10 µM)	128±9	70±5	118±13	58±8
PHS (20 µM)	82±6	22±9	93±8	30±7

SC-galactose liquid media supplemented fatty acids was inoculated with 1×10^5^ cells/ml, incubated for 24 hr at 30°C, and then CFU assay was carried out. The assay was conducted three times.

Abbreviations, 12:0; lauric acid, 16:0; palmitic acid, 18:0; stearic acid, PHS; phytosphingosine.

### Enhanced toxicity of α-syn to aged cells

Because PD and other neurodegenerative diseases increase in frequency with age it is appropriate to explore the relationship between aging and α-syn toxicity. The chronological lifespan (CLS, the survival of stationary phase cells over time) assay is one to way to study aging in yeast [37], [38]. The CLS assay was used to gauge the toxicity of the various α-syns (WT, A30P, A53T and E46K).

**Figure 3:**
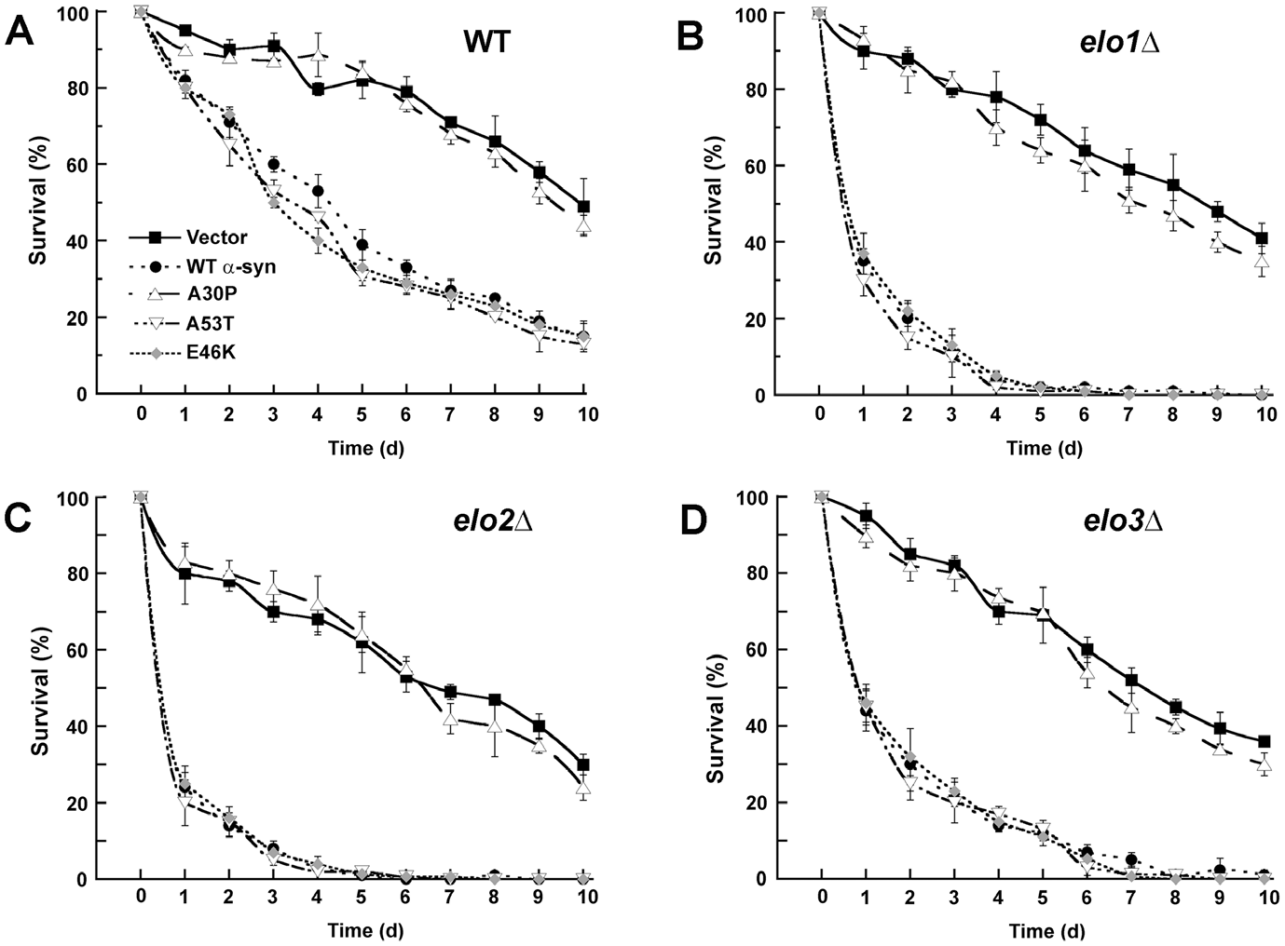
α-Syn is extremely toxic to aged cells. (A) This panel shows chronological aging curves for wild-type BY4741 cells with empty vector (closed square), WT α-syn (closed circle), A30P (open triangle), A53T (inverted open triangle), or E46K (gray diamond). The various cells were grown in selective media containing sucrose to stationary phase, and then the cells were washed and resuspended in selective media containing galactose. After two days of further incubation, the aging analysis started. At the indicated times aliquots were removed form the culture, diluted onto plates containing rich media, incubated for 2 days, and then colonies were counted. Curves represent the number of colonies at the given time divided by the number of colonies at time zero. Experiments were conducted three times, and error bars represent ± s.e.m. P-values are given in the text. Plasmids used: pAG426GAL (empty vector), pAG426GAL-WT (WT α-syn), pAG426GAL-A30P (A30P), pAG426GAL-A53T (A53T), and pAG426GAL-E46K (E46K). (B), (C) and (D) show chronological aging curves for *elo1*Δ, *elo2*Δ, and *elo3*Δ mutants, respectively, with the various α-syns.

Fig. 3 shows the influence of the different α-syns on the chronological life span of various yeast strains. Aging curves show the survival of a population of stationary phase cells over time, and the mean survival time (t_50%_) is the time to 50% survival. The aging curves were almost identical for wild-type cells containing empty vector and expressing A30P (Fig. 3A), i.e., the t_50%_ equaled 12.4±1.3 d and 10.3±0.3 d, respectively. In contrast, WT α-syn, A53T, and E46K were extremely toxic to wild-type cells, decreasing t_50%_ on average to 3.4 days. The enhanced toxicity of WT α-syn to aged cells agrees with a previous study [39]. Aging curves for the various *elo* cells with and without the different α-syns are also shown in Fig. 3, and these aging curves exhibited the following features. First, in experiments using empty vector, each elongase mutant exhibited decreased mean survival compared to wild-type cells (t_50%_ = 12.4±1.3 d). Specifically, for *elo1*Δ, *elo2*Δ and *elo3*Δ cells t_50%_ equaled 8.7±0.4 d (p = 0.022), 6.9±0.4 d (p = 0.013), and 7.5±0.5 d (p = 0.016) (Fig. 3B–D), respectively. On average, an *elo* null mutation produced a 40% decrease in survival compared to wild-type. Second, WT α-syn, A53T and E46K (but not A30P) were extremely toxic to the three elongase mutants, in each case yielding >10-fold decrease in survival (t_50%_: 7-8 d →0.5 d) compared to the same cells with empty vector. On average, α-syn (WT, A53T and E46K) produced a 93% decrease in the mean survival of each null mutant. Third, the assay revealed that α-syn is much more toxic to aged *elo* mutants than to aged wild-type cells. Specifically, the three α-syns (WT, A53T, and E46K) that transit through the secretory pathway and bind to the plasma membrane are extremely toxic to aged elongase mutants, whereas A30P, which does not use the secretory pathway and is predominantly cytosolic, is not.

**Figure 4:**
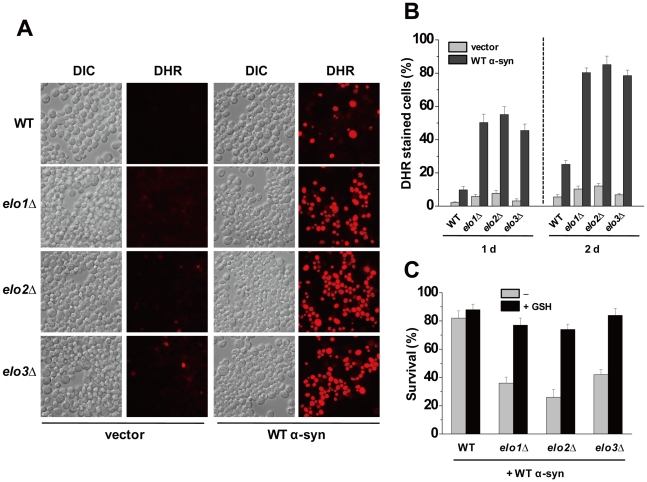
α-Syn triggers ROS in aged *elo* cells. (A) Wild-type cells containing empty plasmid (pAG426GAL) or expressing WT α-syn (pAG426GAL-WT) were cultured for 24 h in inducing media (+gal) at 30°C. After 24 h, cells were incubated with DHR 123 (5 µg/ml) for 1 h, and then visualized by differential interference contrast (DIC) and fluorescence microscopy (DHR). Cells were counted and the percentage of cells exhibiting red fluorescence was plotted. (B) Plot of the percentage of cells (WT, *elo1*Δ, *elo2*Δ, and *elo3*Δ) that exhibited red fluorescence due to ROS accumulation. Cultures were grown for 24 h in inducing media at 30°C and then stained with DHR. Each value was obtained from two independent experiments, where the total number of cells counted was 600. Error bars reflect s.e.m. (C) Reduced glutathione (GSH) protects aged cells from WT α-syn. The various cells expressing WT α-syn were grown in selective media containing sucrose to stationary phase, and then cells were washed and resuspended in galactose media with or without 10 mM GSH. After 3 days (1 day on aging curve) incubation, aliquots were removed from culture, diluted onto plates containing rich media, incubated for 2 days, and then colonies were counted. Each bar represents the number of colonies at the 3 days divided by the number of colonies at time zero. Experiments were conducted three times, and error bars represent ± s.e.m.

α-Syn can trigger reactive oxygen species (ROS) to accumulate in the cytosol of human cells and yeast cells and this oxidant overload may contribute to cell death by apoptosis [40], [41]. An issue is whether ROS accumulates during aging, and for this purpose the cell permeable dye dihydrorhodamine 123 (DHR 123), which fluoresces only when oxidized, was used. We found that in the absence of α-syn expression *elo* cells and wild-type cells exhibited negligible ROS (<10% of cells stained red) after 24 h (Fig. 4A). In contrast, *elo* cells expressing WT α-syn accumulated appreciable ROS after 24 h compared to the same cells with empty vector (Fig. 4A), i.e., ∼50% versus ∼5% of *elo* cells stained for ROS with and without WT α-syn expression (Fig. 4B), respectively. After 48 h, the differences in ROS accumulation between the aged *elo* cells with or without α-syn expression were even more striking. The results show that reactions catalyzed by the Elo enzymes—the formation of C26 fatty acid and C26-ceramide—are necessary to suppress α-syn-induced ROS accumulation, and that the rapid decrease in survival in the aged *elo* mutants expressing WT α-syn (Figure 3B–C) correlates with the massive accumulation of ROS induced by α-syn.

If ROS accumulation causes the rapid die-off of aged *elo* cells expressing WT α-syn, then an anti-oxidant such as glutathione (GSH), which effectively eliminates ROS caused by WT α-syn [41], should inhibit ROS accumulation and prevent the rapid loss of viability. Figure 4C shows data obtained from 1-d old aged cells with or without GSH. GSH strongly protected the various aged *el*o mutants from WT α-syn, as evidenced by the large (≥100%) increases in survival compared to aged *elo* cells expressing WT α-syn without GSH. On the basis of these results, we conclude that ROS accumulation induced by (WT, A53T or E46K) α-syn causes the rapid die-off of aged cells, particularly aged *elo* cells.

**Figure 5:**
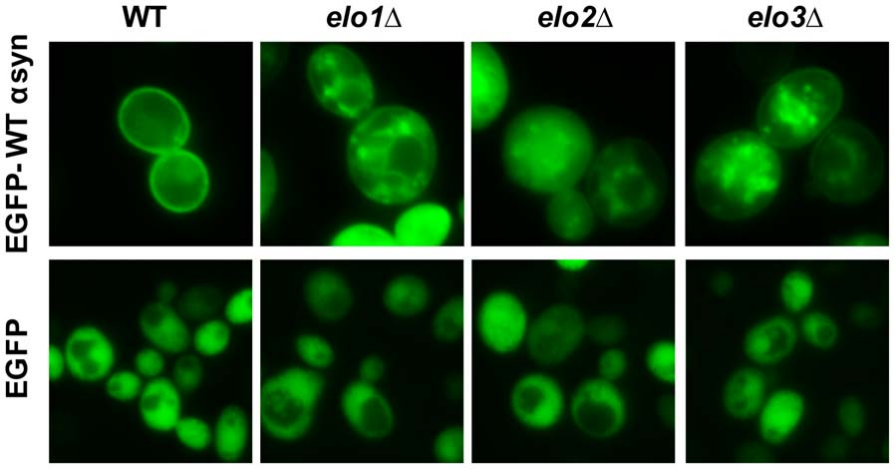
Elongase null mutations alter the localization of WT α-syn. Yeast cells (BY4741, *elo1*Δ, *elo2*Δ, and *elo3*Δ) expressing eGFP-WT-α-syn or EGFP were cultured in inducing media for 6 h at 30°C and then visualized by fluorescence microscopy. Plasmids: pAG426GAL-EGFP-WT-α-syn and pAG426GAL-EGFP.

To determine whether the *elo* null mutations perturb the localization of WT α-syn, fluorescence microscopy was conducted using a green fluorescent variant of WT α-syn (EGFP-WT-α-syn) after a 6 h induction. In wild-type cells, EGFP-WT-α-syn primarily localized to the plasma membrane (Fig. 5); whereas, in *elo1*Δ cells, EGFP-WT-α-syn accumulated in multiple cytosolic inclusions or vesicles per cell, and, in general, less plasma membrane binding occurred. Similar results were obtained for *elo2*Δ and *elo3*Δ cells. EGFP showed the same diffuse cytoplasmic localization in the various strains. Loss of the various Elo enzymes clearly affects the localization of α-syn, and the altered localization might underlie the enhanced toxicity of α-syn these strains.

### Cells expressing α-syn (WT, A53T or E46K) are sensitive to fumonisin B1 (FB1) and myriocin

The elongase enzymes Elo2p and Elo3p are required for the synthesis of C26-fatty acids, which is a component of ceramides and sphingolipids, and loss of either of these two enzymes results in ceramides that contain fatty acids other than the required C26 FA [31], [32]. In yeast, two enzymes, Lag1p and Lac1p, synthesize ceramides [42], [43], [44]. We tested *lag1*Δ and *lac1*Δ strains for sensitivity to α-syn but found no effect (Table 2). We also tested a *lag1*Δ*lac1*Δ double deletion strain for sensitivity to α-syn, but had difficulty growing the strain in galactose; thus, it could not be determined whether elimination of ceramide synthase activity increased α-syn toxicity. As an alternative approach, we depleted cells of ceramide using FB1 [45] to inhibit ceramide synthase or myriocin to inhibit serine palmitoyltransferase (SPT) [46].

**Figure 6:**
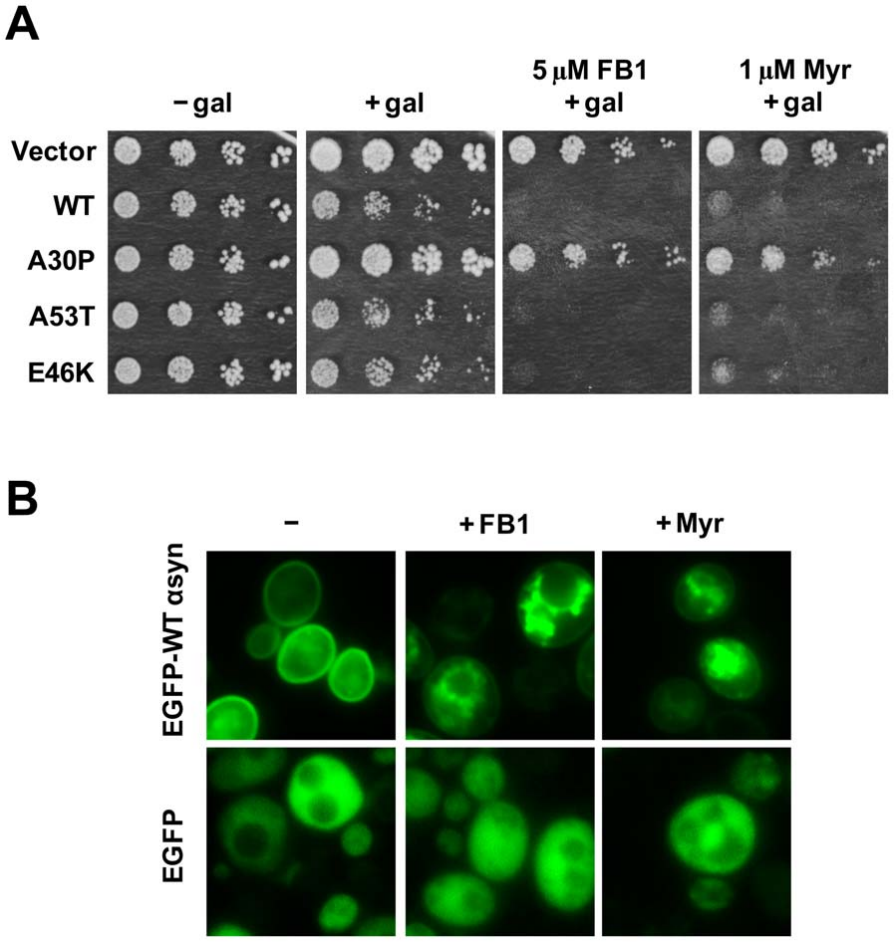
Hypersensitivity of cells expressing α-syn (WT, A53T or E46K) to fumonisin B1 (FB1) and myriocin. (A) The toxicity of FB1 and myriocin was evaluated by evaluating the growth properties of various cells in a serial dilution assay. Strains were transformed with empty vector or plasmids harboring the various α-syns (WT α-syn, A30P, A53T, or E46K) and spotted in successive 10-fold serial dilutions on solid sucrose and solid galactose plates and incubated for 3 days at 30°C. FB1 and myriocin were dissolved in the media at 5 µM or 1 µM, respectively. Experiments were repeated three times with similar results. The pAG426GAL set of plasmids were used. (B) FB1 and myriocin drives WT α-syn off the plasma membrane and into inclusions. Wild-type cells expressing eGFP-WT-α-syn or eGFP were incubated for 6 h in inducing media containing 5 µM FB1 or 1 µM myriocin or drug vehicle, ethanol (−) at 30°C. Images were obtained by fluorescence microscopy 6 h after adding myriocin. Approximately 85%, and 80% of the cells examined exhibited the EGFP-WT-α-syn inclusions as shown in the upper center (+Fum) and right panel (+Myr), respectively. Plasmids: pAG426GAL-EGFP-WT-α-syn and pAG426GAL-EGFP.

The growth properties of BY4741 cells transformed with vector control or various α-syns (WT, A30P, A53T or E46K) and with FB1 (5 µM) or myriocin (1 µM) added to the galactose plates is shown in Fig. 6A. FB1 and myriocin were extremely toxic to cells expressing WT α-syn, A53T or E46K compared to vector control cells, but much less toxic to cells expressing A30P. Each compound also slowed the growth of the cells with empty vector, but the effects were far more pronounced in the cells that expressed the α-syns that transit through the secretory pathway.

Fluorescence microscopy revealed the effects of the two inhibitors on the localization of EGFP-WT-α-syn in wild-type cells. In cells without added drugs, EGFP-WT-α-syn was clearly enriched in the plasma membrane (Fig. 6B), whereas EGFP was distributed through out the cells. In cells expressing GFP-WT-α-syn and treated with FB1 the GFP-WT-α-syn protein was nearly absent from the plasma membrane; instead, the protein localized in cytosolic inclusions, vesicles, or a cellular compartment. Similar images were obtained for cells expressing GFP-WT-α-syn and treated with myriocin. Both drugs failed to affect the localization of EGFP. The results indicate that cells that fail to synthesize ceramides have defects in the trafficking of α-syn.

### A plasma membrane-binding form of EGFP is toxic, like α-syn, to *elo3*Δ cells

To probe protein trafficking to the plasma membrane in *elo* cells, two different variants of EGFP that contain a short C-terminal plasma membrane-targeting sequence were constructed: EGFP-mts1 (mts1, SNSVCCTLM) contains a short C-terminal membrane targeting sequence derived from Ste18 protein, and EGFP-mts2 (mts2, GSGGCCIIS) contains a similar C-terminal sequence derived from Ras2 [47]. Ste18 traffics through the ER to the plasma membrane whereas Ras2 may use an alternative pathway en route to the plasma membrane. Targeting a non-toxic, universally used protein like EGFP to the plasma membrane (by whatever route) could give insight into the molecular defects in *elo3*Δ cells. Below, wild-type cells expressing the different EGFP variants (with or without added FB1) are compared to *elo3*Δ cells expressing the different EGFP variants.

**Figure 7:**
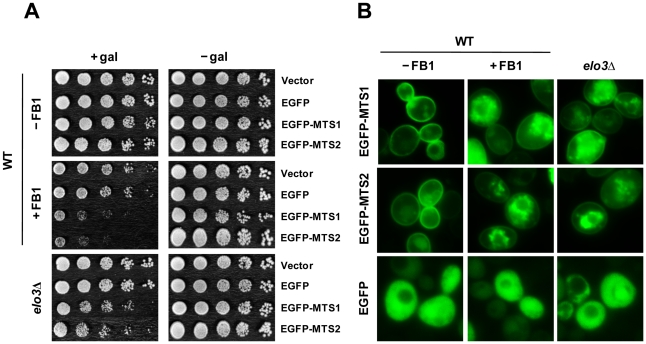
A plasma membrane-binding form of EGFP is toxic, like α-syn, to *elo3*Δ cells. (A) Inhibiting ceramide synthetase with FB1, serine palmitoyltransferase with myriocin or blocking C26-VLCFA synthesis in *elo3*Δ cells drives EGFP-mts1 and EGFP-mts2 into cytoplasmic inclusions. Wild-type cells or *elo3*Δ cells expressing EGFP-mts1, EGFP-mts2 or EGFP as a control were incubated for 6 h in inducing media containing 5 µM FB1, 1 µM myriocin (or drug vehicle, ethanol) and then imaged by fluorescence microscopy. Approximately 80%, 68%, and 88% of the cells examined exhibited the EGFP-mts1 inclusions as shown in the wild-type strain (+Fum), (+Myr), and in the *elo3*Δ strain, respectively. 85%, 75%, 70% of the cells examined exhibited the EGFP-mts2 inclusion as shown in the wild-type strain (+Fum), (+Myr), and in the *elo3*Δ strain, respectively. Plasmids: pAG426GAL-EGFP-mts1, pAG426GAL-EGFP-mts2, and pAG426GAL-EGFP. (B) Growth properties of cells expressing EGFP-mts1, EGFP-mts2, or EGFP were evaluated in a dilution spot assay. EGFP-mts1 or EGFP-mts2 is not toxic to wild-type cells, whereas it was very toxic to FB1-treated (5 µM) wild-type cells and to *elo3*Δ cells. The control construct EGFP was not toxic to any of the cells. The assay was conducted according to the legend in Fig. 1.

The growth properties of cells expressing EGFP-mts(1,2) or EGFP are shown in Fig. 7A. EGFP-mts(1,2) and EGFP were not toxic to wild-type cells, as judged by the lack of growth defects compared to vector control cells. However, when the same cells were treated with FB1 (5 µM) severe growth defects occurred for cells expressing EGFP-mts(1,2) but not for cells expressing EGFP. Inhibiting ceramide synthesis clearly had a much bigger effect on cells expressing the mts-tagged EGFP molecules. Growth properties of *elo3*Δ cells expressing EGFP-mts(1,2) or EGFP were also conducted. In this mutant, which synthesizes C24-ceramides rather than C26-ceramides [31], [32], slight but reproducible growth defects occurred for cells expressing the mts-tagged EGFP molecules. The data show that inhibiting ceramide synthesis or synthesizing ceramides with slightly shorter fatty acid chains produces growth defects in cells that express EGFP with a plasma membrane targeting sequence.

Fluorescence microscopy was conducted to assess the effects of FB1 and *elo3*Δ on EGFP (± mts tag) localization (Fig. 7B). The EGFP-mts1 protein localized to the plasma membrane, as expected, whereas the untagged variant was distributed through out cells. The same cells treated with FB1 had much less membrane bound EGFP-mts1; instead, the protein was clustered in the cytosol in inclusions, vesicles, or in some cellular compartment. Similar results were obtained for cells expressing EGFP-mts2. Note that *elo3*Δ cells expressing EGFP-mts(1,2) also had much less EGFP-mts(1,2) on the plasma membrane than wild-type cells, and overall, in this mutant the membrane-tagged EGFP molecules sequestered in the cytosol in inclusions, vesicles, or in some cellular compartment. Inhibiting ceramide synthesis or slightly altering the length of the fatty acid chain has dramatic effects on the trafficking of the EGFP-mts molecules through the cell.

## Discussion

We have shown that the *elo* mutations and ceramide synthesis inhibitors dramatically enhance the toxicity of those α-syn variants that use the classical secretory pathway, i.e., WT, A53T, and E46K α-syn. Whereas, A30P, which does not use the classical secretory pathway, was unaffected by such mutations and inhibitors. Overall, this study shows that disruption of lipid homeostasis in the ER converts WT α-syn from a relatively non-toxic protein into a toxic one. Humans possess six Elo genes (ELOVLs) [48], and these genes are gaining attention because of their roles in diabetes [49], macular degeneration [50], cancer [51], and the production of long chain polyunsaturated fatty acids [52].

### Enhanced α-syn toxicity in *elo1*Δ

The *elo1*Δ mutant has the same lipid profile as wild-type cells [26], but, in nearly all our assays, the *elo1*Δ mutant and wild-type cells were different (Figs. 1, 2, 3, 4, 5). One possibility is that a different distribution of lipids occurs in the two strains because Elo1p and FAS carry out the same reaction (C14 fatty acid→ C16 fatty acid) but in different compartments. To compensate for the loss of Elo1p activity, palmitoyl-CoA must transfer from the cytosol into the ER, and if this transfer is inefficient mutant cells will have a sub-optimal concentration of this important lipid in the ER (and excess in the cytosol). The defects associated with *elo1*Δ cells observed in this study could be due to a deficit of palmitoyl-CoA in the ER compared to wild-type cells. Furthermore, because the ER is the compartment where proteins are palmitoylated, a process that requires palmitoyl-CoA, perhaps *elo1*Δ cells are defective in protein palmitoylation. We propose that α-syn (WT, A53T, or E46K) is more toxic to *elo1*Δ cells than to wild-type cells because *elo1*Δ cells expressing α-syn probably have two ER defects, i.e., one from the loss of palmitate and the other because α-syn inhibits ER to Golgi traffic [21]. WT α-syn is even more toxic to aged *elo1*Δ cells as evidenced by massive ROS accumulation (Figs. 3, 4).

Depending on the concentration, exogenous palmitate (C16) was helpful or harmful to wild-type and *elo1*Δ cells expressing WT α-syn (Table 3). In general, 10 mM palmitic acid was toxic to wild-type and *elo1*Δ cells expressing WT α-syn compared to 1 mM palmitic acid. Over-expressing Elop1 from a 2 µ plasmid was also very toxic to cells expressing WT α-syn (Fig. 2B), which indicates that elevated palmitic acid enhances α-syn toxicity. Note that prolonged exposure of α-syn to the lipid docosahexaenoic acid, which occurs at high levels in the brain, induces α-syn to form amyloid fibers [53]. Based on our findings, we propose that the toxicity of WT α-syn exhibits a U-shaped toxicity profile against palmitate and other lipids/sphingolipids.

### Sphingolipid metabolism defects in *elo2*Δ and *elo3*Δ cells

Biochemical analysis of lipid extracts from the *elo2*Δ and *elo3*Δ mutants have shown that these mutants fail to synthesize VLCFAs and have defects in sphingolipid metabolism [27], [54]. In the case of *elo3*Δ, cells fail to synthesize C26 VLCFA and C26-phytoceramide, instead they synthesize C22- and C24- forms of phytoceramide [54]. Aged *elo2*Δ and *elo3*Δ mutants exhibited a modest, but significant, decrease in survival compared to aged wild-type cells, although neither mutant accumulated significant levels of ROS (Figs. 3, 4). α-Syn was extremely toxic to aged *elo2*Δ and *elo3*Δ cells compared to aged wild-type cells. Specifically, α-syn triggered massive ROS accumulation, which triggers the precipitous decrease in survival (Figs. 3, 4). Note that the membrane-targeting forms of EGFP were even slightly toxic to *elo3*Δ cells (Fig. 7). The *elo3*Δ mutant is known to cause the aberrant trafficking of the essential plasma membrane proton pump protein Pma1 [32]. For example, instead of transiting through the ER to the plasma membrane Pma1 targets to the vacuole for degradation in *elo3*Δ cells. We propose that the *elo2*Δ and *elo3*Δ mutants have subtly altered ceramide/sphingolipid homeostasis that in turn triggers protein trafficking defects in the ER. Expressing α-syn in these mutant cells, especially aged cells, exacerbates these trafficking defects and leads to aberrant α-syn trafficking, ROS accumulation and cell death.

### Inhibition of ceramide synthesis

Fumonisin B1 is an environmental toxin synthesized by various fungi that infest maize (corn) worldwide (see: www.inchem.org/documents/ehc/ehc/ehc219.htm). Pregnant woman who consume FB1-infested corn show a higher incidence of children born with neural tube defects [55], [56]. Consumed at high levels over a long period of time (perhaps decades) FB1 may produce neuropathological effects in humans. The point is that this toxin can alter α-syn trafficking and boost its toxicity, as shown herein.

Fumonisin B1 and myriocin were extremely toxic to cells expressing WT, A53T or E46K α-syn but much less toxic to cells expressing A30P (Fig. 6A). These drugs were also extremely toxic to cells expressing membrane-targeting forms of EGFP but not soluble EGFP (Fig. 7A). Both drugs appeared to prevent the trafficking of WT α-syn (and EGFP-mts) to the plasma membrane (Figs. 6B, 7B). Our interpretation of these findings is that these two drugs disrupt the ceramide-sphingolipid homeostasis in the ER and this causes aberrant trafficking of proteins that transit through the ER like WT, A53T or E46K α-syn. The combination of α-syn aberrant trafficking, α-syn inclusion formation and ROS accumulation kills cells.

Given our findings of the sensitivity of α-syn trafficking to the proper lipid homeostasis in the ER, we ask whether a decrease in VLCFA or sphingolipid levels might occur with age in some people, and whether such a condition might predispose one to PD. Intriguingly, results from a 9 year longitudinal study of 100 women showed that serum levels of sphingomyelins (SM) and ceramide may be good pre-clinical biomarker of memory impairment like that associated with the long preclinical stage of Alzheimer's disease [57]. In short, it is reasonable to investigate whether a low level of VLCFAs or ceramide is a risk factor for PD. If so, these compounds could serve as biomarkers for PD.

## Materials and Methods

### Yeast strains, media, and transformation

The primary yeast strains used in this study were BY4741 (American Type Culture Collection; ATCC, Manassas, VA), and BY4741-ELO1-TAP (Open Biosystems), which contains an integrated copy of *ELO1-TAP* replacing the WT allele (Table 1). Deletion strains derived from haploid yeast BY4741 and S288C were purchased from ATCC or were a gift from Kelly Tatchell (LSUHSC, Shreveport, LA), respectively. Rich medium consisted of 1% yeast extract, 2% peptone, and 2% glucose (YPD). Synthetic complete (SC) medium consisted of 0.67% yeast nitrogen base, 0.16% yeast drop-out mix (Sigma-Aldrich, St-Louis, MO), and carbon sources (2% glucose, sucrose, or galactose). Plates contained 2% agar (Difco, Detroit, MI). Transformation of yeast strains was performed by the lithium acetate method [58]. Cells transformed with various plasmids were pre-grown in SC-glucose drop out media to maintain selection for plasmids. Typically, in the various experiments, cells harboring the plasmid of interest were pre-grown until mid-log phase in SC-sucrose drop out media (non-inducing media) and then centrifuged, washed and resuspended in SC-galactose drop out media (inducing media). In some cases, media was supplemented with fatty acids, i.e., 0.5% Brij 58 and C12:0, C16:0, or C18:0 fatty acids (Sigma) at concentrations of 1 mM or 10 mM. Phytosphingosine (PHS) was dissolved in ethanol (at 10 mM) and added to sterilized culture media to yield final concentration of 5 or 10 µM. FB1 and myriocin (Sigma) were used as a 2.5 mM stock solution in ethanol and added to sterilized media to a final concentration of 1 or 5 µM.

### Plasmid construction

The plasmids used in this study are listed in Table 1. Plasmids were constructed using the *in vitro* recombination-based Gateway cloning system (Invitrogen, Carlsbad, CA) (as described in [59]). The coding sequence of α-syn (WT, A30P, A53T, or E46K) (from template plasmids [41]) and *ELO1* (from BY4741 genomic DNA) was amplified by PCR with forward and reverse primers that contained a 25-nucleotide (nt) *att*B sequence and 25-30 nt of the complementary gene-specific sequence. Purified double-stranded PCR products were then recombined with the counter-selectable *ccd*B gene of the donor vector pDONR221 using the BP clonase enzyme mixture. The donor vector containing the gene of interest was then cloned into the Gateway destination vectors pAG426GAL, pAG426GAL-GFP, pAG415GPD, pAG415GPD-TAP, pAG425GAL, or pAG425GAL-TAP (Addgene, Cambridge, MA) using the LR clonase enzyme mixture. The pDONR221 donor vector is commercially available (Invitrogen).

The EGFP-plasma membrane targeting sequence of Ste18p (GFP-mts1) and EGFP-plasma membrane targeting sequence of Ras2p (GFP-mts2) constructs were also prepared using the recombination-based Gateway cloning system. The EGFP inserts from a template plasmid were PCR amplified using the forward primer 5'-GGGGACAAGTTTGTACA-AAAAAGCAGGCTTCGAAGGAGATAGAACCATGAGTAAAGGAGAAGAACTTTTCACTG-3′, which contains the *attB1* sequence and a sequence complementary to the DNA coding for EGFP, and the reverse primers 5′-GGGGACCACTTTGTACAAGAAAGCTGGGTCCTACATAAGCGTACAACAAACAC TATTTGATTTGTATAGTTCATCCATGCCATGTG-3′; which contains the *attB2* sequence and a sequence that codes for the plasma membrane targeting sequence (mts1 protein sequence: SNSVCCTLM) and 5′-GGGGACCACTTTGTACAAGAAAGCTGGGTCCTAACTTATAATACAACAGCCACCCGATCCTTTGTATAGTTCATCCATGCCATGTG-3′, which contains the *attB2* sequence and a sequence that codes for the plasma membrane targeting sequences (mts2 protein sequence: GSGGCCIIS), respectively. The purified double-stranded PCR products were transferred into the Gateway destination vector pAG426GAL yielding pAG426GAL-EGFP-mts1 and pAG426GAL-EGFP-mts2 in the sequence of steps described above, respectively.

### Growth and CFU assay

For analysis of growth by plate assays, yeast cells grown in 3 ml of SC-sucrose media in 16-mm sterilized tubes with plastic cap for overnight (15 h) at 30°C were harvested by centrifuge (7000×g) for 10 minutes at room temperature, washed with phosphate buffered-saline (PBS), and resuspended in the same buffer to a concentration of 1.0×10^8^ cells ml^-1^. After the cell suspensions were serially diluted in 10-fold steps, a 5 µl aliquot of each dilution was spotted onto SC-sucrose (-gal) and SC-galactose (+gal) plates, and the plates were then incubated for 3 days at 30°C.

For growth assay in the liquid medium, colony forming units (CFU) assay was performed. Aliquots of the washed cell suspension were inoculated into 5 ml of SC-galactose or with fatty acids and long chain base (PHS) media at final concentration of 1.0×10^5^ cells ml^-1^. 10 µl aliquots of the 5 ml cultures were removed at the indicated time-points, diluted in sterile PBS buffer, spread onto at least three YPD plates (maximum colony ∼500 colonies per plate), and allowed to grow into colonies for 3 days. The colonies were then counted.

### Chronological life span assay

Chronological aging experiments were performed as described [60], [61]. Yeast cells were inoculated into 5 ml of SC-sucrose media in 18-mm glass culture tubes with plastic caps. These starter cultures were grown overnight on a rotating roller drum (model TC-7; New Brunswick Scientific, Edison, NJ, USA) such that the tubes were vertically tilted ∼15° from horizontal and rotating at ∼50 rpm to maintain the cells in suspension. The roller drum was positioned within an incubator set at 30°C. One hundred microliters of the overnight culture was then inoculated into 5 ml SC-galactose media and incubated on the roller drum for the duration of the experiment, except for brief times that cultures were removed for plating of aliquots. Forty-eight hours later was considered as the zero time-point (day 0). Cell viability on aging was measured by CFU assay as listed above.

### Western blot analysis

Western blots were conducted as described [41], with minor modification. In brief, yeast cells transformed with plasmids with α-syn (pAG426GAL-WT, A30P, A53T, or E46K) and Elo1p-TAP (pAG415GPD-ELO1-TAP or pAG425GAL-ELO1-TAP) or the Open Biosystems ELO1-TAP-tagged integrated strain were cultured in SC-sucrose media to mid-log phase and then shifted into SC-galactose media for 6 h at 30°C. Cells were lysed (and all subsequent manipulations were carried out at ice temperature), a protease inhibitor cocktail (Roche) was added, and then the protein concentration was determined (Bio-Rad protein assay). Samples (80 µg/well) were subjected to SDS-PAGE and then transferred to a nitrocellulose membrane for detection of α-syn and TAP. The monoclonal antibody against human α-syn was purchased from Cell Signaling Technologies (#2642). The monoclonal antibody against Pgk1p was purchased from Invitrogen. The polyclonal antibody against TAP was purchased from Open Biosystems. Secondary antibodies were purchased from Santa Cruz Biotechnology. Chemoluminescence detection (GE Health Care) was used to visualize α-syn, TAP, and Pgk1p. Western blot analysis was repeated three times.

### Fluorescence microscopy

Fluorescence microscopy was performed with an Olympus AX70 microscope, equipped with an Olympus UPlanFI 100×/1.35 NA objective and a Roper CoolSNAP HQ CCD camera. The acquisition software was IPLab v.3.6 from Scanalytics Inc. Chroma 41001 (HQ 480/40 exciter, HQ 535/50 emitter, Q 505 LP dichroic) filter set was used for detecting EGFP. Data were collected at room temperature. The ROS assay, using the DHR 123 dye, was performed as described previously [41]. Aged cells for 1 d were stained with DHR 123 (5 µg/ml) for 1 h at 30°C.

### Error analysis

The t_50_ values from the various *elo* (± α-syn) aging curves were compared to control curves with a paired Student's t-test using EXCEL.
